# Vaccines for Pandemic Influenza

**DOI:** 10.3201/eid1201.051147

**Published:** 2006-01

**Authors:** Catherine J. Luke, Kanta Subbarao

**Affiliations:** * National Institutes of Health, Bethesda, Maryland, USA

## Abstract

Recent outbreaks of highly pathogenic avian influenza in Asia and associated human infections have led to a heightened level of awareness and preparation for a possible influenza pandemic. Vaccination is the best option by which spread of a pandemic virus could be prevented and severity of disease reduced. Production of live attenuated and inactivated vaccine seed viruses against avian influenza viruses, which have the potential to cause pandemics, and their testing in preclinical studies and clinical trials will establish the principles and ensure manufacturing experience that will be critical in the event of the emergence of such a virus into the human population. Studies of such vaccines will also add to our understanding of the biology of avian influenza viruses and their behavior in mammalian hosts.

## Abstract 2

A program to develop vaccines to prevent avian influenza pandemics is under way.Vaccines for Pandemic Influenza

Influenza is a negative-strand RNA virus that belongs to the family *Orthomyxoviridae*, which consists of 4 genera: influenza A, influenza B, influenza C, and Thogoto viruses. The proteins of influenza A are encoded on 8 RNA gene segments. Influenza A viruses are widely distributed in nature and can infect a wide variety of birds and mammals. Influenza A virus subtypes are classified on the basis of the antigenicity of their surface glycoproteins, hemagglutinin (HA) and neuraminidase (NA); 16 HA and 9 NA subtypes are known to exist, and all of them infect aquatic birds. Most infections in waterfowl are not associated with clinical disease. Relatively few subtypes of influenza A viruses have caused sustained outbreaks of disease in the human population. Influenza A viruses of the H1, H2, and H3 HA and of the N1 and N2 NA subtypes have circulated in the human population in the 20th century. H1N1 viruses appeared in 1918 and circulated until 1957, when they were replaced by H2N2 viruses. H3N2 viruses appeared in 1968, replacing H2N2 viruses, and have remained in circulation in the human population. H1N1 viruses reappeared in the human population in 1977 and continue to cocirculate with H3N2 viruses (*1*). Currently, influenza epidemics in the winter are caused by H3N2 and H1N1 influenza A and influenza B viruses.

## Influenza Pandemics

In addition to seasonal influenza epidemics, influenza pandemics have occurred periodically. An influenza pandemic occurs when an influenza strain with a novel HA subtype (with or without a novel NA subtype) appears and spreads in the human population, which has little or no immunity to the novel HA. In the 20th century, pandemics occurred in 1918, 1957, and 1968 and were associated with substantial illness and death. The pandemic of 1918, the "Spanish flu," was caused by an influenza A virus of the H1N1 subtype and was responsible for >40 million deaths worldwide (*2*). In the Asian influenza pandemic of 1957, in which H2N2 viruses appeared, influenza-associated excess deaths were estimated at >2 million worldwide (*3*). The influenza pandemic of 1968 started in Hong Kong and was caused by an H3N2 virus. The 1968 pandemic virus had the same NA as the H2N2 virus it replaced but a novel HA. This pandemic was much less severe than the previous pandemics, with estimated influenza-associated excess deaths of ≈1 million (*3*). Preexisting immunity to the N2 NA of the 1968 pandemic influenza virus may partially explain why this pandemic was less severe than the 2 preceding pandemics, although the availability of penicillins and macrolides may also have contributed.

We cannot predict when the next influenza pandemic will occur, or which influenza virus subtype will cause it. Forecasts of the severity of the next influenza pandemic differ in their predictions of deaths based on the models used. Modeling based on the pandemic of 1968 projects 2 million–7.4 million excess deaths worldwide (*3*). Meltzer and colleagues have estimated that, in the absence of effective interventions, in the United States alone, the next influenza pandemic could cause 89,000–207,000 excess deaths and 314,000–734,000 hospitalizations, as well as tens of millions of outpatient visits and additional illnesses (*4*). In this scenario, the economic impact of an influenza pandemic would be severe. The economic costs due to deaths, illness, and hospitalizations in the United States alone, excluding disruptions to commerce and society, would be $71.3–$166.5 billion (*4*).

In the recent H5N1 outbreaks in Asia, >120 million birds died or were culled during a 3-month period (*3*). For the countries of Thailand and Vietnam, the estimated decreases in gross domestic product (GDP) resulting from poultry farming losses are $1.2 and $0.3 billion, respectively, with a total estimated loss in GDP for Asia of $10 to $15 billion, according to the same source. In addition to the effects on local poultry production and commerce, international trade and travel would undoubtedly suffer in an influenza pandemic.

### Avian Species as a Reservoir for Pandemic Influenza Viruses

Aquatic birds are the reservoir for all known subtypes of influenza A viruses, and as such are the pool from which pandemic influenza viruses arise. Avian influenza (AI) viruses are introduced into the human population after reassortment with circulating human influenza A viruses or by directly infecting humans.

Until 1997, it was widely believed that to infect humans an AI virus would have to undergo reassortment with a human influenza virus in an intermediate mammalian species to acquire the necessary characteristics for efficient transmission to and replication in humans. In the last 10 years, direct transmission of AI viruses from birds to humans has been reported on several occasions, causing a wide spectrum of disease, ranging from mild febrile and respiratory illness in some H5 and H9N2 infections, conjunctivitis in the case of H7 influenza infections, to severe disease and death, as seen in the current H5N1 outbreak in Asia. The details of these cases are given in Table 1.

**Table 1:** Direct transmission of avian influenza viruses to humans

Virus subtype	Year	Location	No. cases (no. deaths)	Clinical features	Notes	Reference(s)
H5N1	1997	Hong Kong	18 (6)		Associated with outbreak of highly pathogenic AI in poultry in the region	(*5**,**6*)
H9N2	1999	Hong Kong	2 (0)	Mild influenzalike illness		(*7*)
H9N2	1999	Guangdong Province, China	5 (0)	Mild influenzalike illness		(*8*)
H9N2	2003	Hong Kong	1 (0)	Mild influenzalike illness		(*9*)
H5N1	2003	Hong Kong	2 (1)	Primary viral pneumonia, lymphopenia, respiratory distress	7-year-old girl died in Fujian Province, China, and H5N1 infection was not confirmed. Her 33-year-old father died from confirmed H5N1 influenza infection in Hong Kong, and her 8-year-old brother recovered from H5N1 infection.	(*10*)
H7N7	2003	Netherlands	89 (1)	Conjunctivitis (78 cases), mild influenzalike symptoms (2 cases) or both (5 cases). In fatal case, pneumonia followed by respiratory distress syndrome	Most cases were in persons involved in handling poultry (86), with 3 family members also affected.	(*11*)
H10N7	2004	Egypt	2 (0)	Fever and cough	Both cases were in infants, who recovered without complications	(*12*)
H5N1	2003–present	Asia (Vietnam, Thailand, Cambodia, Indonesia)	116 (60)*	Fever, respiratory symptoms, lymphopenia, elevated liver enzymes. Severe cases progess to respiratory failure, multiple organ dysfunction, and death.	Human cases concomitant with unprecedented outbreaks of highly pathogenic H5N1 AI in poultry	WHO,* (*13**–**15*)

*WHO, World Health Organization. As of September 29, 2005. Source: http://www.who.int/csr/disease/avian_influenza/country/en

The gene segments of the influenza viruses isolated from the human H5N1 patients in 1997 were all derived from AI viruses, with no evidence of reassortment with human influenza viruses. Surveillance studies in birds in Hong Kong showed that H5N1 and H9N2 AI viruses cocirculated in poultry markets in Hong Kong at the time of the 1997 H5N1 AI outbreak, creating favorable conditions for reassortment (*16*). H9N2 AI viruses had become widespread in domestic chickens in Asia since 1990. In addition, both of these AI subtypes were isolated from ducks and geese in the region, suggesting a wide distribution in avian hosts. Data from phylogenetic studies led to the hypothesis that the H5N1 Hong Kong viruses acquired their HA gene from an A/goose/Guangdong/1/96-like (H5N1) virus and the gene encoding NA from an A/teal/Hong Kong/W312/97 (H6N1)-like virus circulating in terrestrial poultry. Data also suggested that H9N2 or H6N1 AI viruses circulating in the region were the likely source of the internal protein genes (*17**–**20*). H9N2 viruses continue to circulate in birds in southern China.

The outbreak of human H5N1 cases in 1997 ended with the depopulation of the poultry markets in Hong Kong. These actions may have averted an influenza pandemic (*16*). Precursor viruses, however, continued to circulate in the region, and in 2003, highly pathogenic H5N1 viruses reemerged, and new human infections were identified and continue to be reported to date.

### Preparing for the Next Pandemic

The reemergence of highly pathogenic H5N1 AI viruses in Asia has raised concerns of a potential pandemic, resulting in an augmented level of preparedness for such an eventuality. The pandemic preparedness plan for the United States was published in November 2005 (http://www.hhs.gov/pandemicflu/plan/).

Two intervention strategies could prevent or lessen the severity of an emergent influenza pandemic, vaccination and use of antiviral drugs. The use of antiviral compounds is discussed in another article in this issue (*21*). We focus on the challenges facing development of pandemic influenza vaccines and how we can prepare and test a library of vaccine seed viruses. Although the next influenza pandemic could possibly be caused by a different avian or reassortant virus than the highly pathogenic H5N1 AI virus now circulating in Asia, current vaccine development activities are largely focused on viruses of this subtype. Events in Asia underscore the urgent need for generating candidate H5N1 vaccines and evaluating them in humans, but ignoring AI viruses of the other subtypes would be imprudent. All AI viruses are presumed to have pandemic potential.

## Developing Vaccines for Pandemic Influenza

Central to pandemic preparedness planning are effective vaccines to thwart the spread of a pandemic virus and to prevent illness and death associated with a novel virulent strain. The principle behind the generation of human influenza vaccines is to elicit protective antibodies directed primarily against HA, the major protective antigen of the virus that induces neutralizing antibodies. Although major advances in our understanding of the biology and ecology of the H5N1 AI viruses have been made since human infections were first reported in 1997, and we have many years of experience and much accumulated knowledge about immunity to human influenza viruses, gaps remain in our understanding of immunity to AI viruses (Table 2). Filling in these gaps is vital to developing vaccines to protect the human population. Studies using inactivated vaccines against H9N2 and H5 subtypes of AI or purified recombinant H5 HA have demonstrated that these vaccines are poorly immunogenic in comparison to epidemic human influenza strains of the H1N1 and H3N2 subtypes. For example, inactivated vaccines against avian influenza subtypes require 2 doses and administration with adjuvant to achieve the desired level of neutralizing antibody (*22**–**27*) (Table 3). The precise antigenic properties of a nascent pandemic strain cannot be predicted, so available vaccines may be poorly antigenically matched to the pandemic virus. Practical considerations and hurdles for pandemic influenza vaccine development also have to be overcome. Manufacturing capacity, the ability of candidate vaccine strains to grow well in eggs, and biological safety containment of parent strains for vaccine development are all problems to be addressed. In addition, the most vulnerable sections of the population may not be the same as those seen with yearly influenza epidemics, making planning to target certain population groups for vaccination difficult at best. For these reasons, the time before the next pandemic must be used judiciously for developing and clinically testing candidate vaccines.

**Table 2:** Challenges for developing vaccines for pandemic influenza: knowns and unknowns*

What we know from experience with human influenza viruses	What we don’t know
Antibodies against the HA (and to a lesser extent NA) are critical for protection. Systemic immune response is strain specific. Mucosal immune response provides broader cross-protection. Cellular immunity is needed for viral clearance. Vaccine strain must closely match the circulating strain.	Which avian influenza virus will cross species barrier to cause a pandemic Importance of antigenic drift among avian influenza viruses Immunogenicity of HA of avian viruses in humans (unknown or poor)

*HA, hemagglutinin; NA, neuraminidase.

**Table 3:** Details of clinical trials in humans of inactivated and subunit vaccines against avian influenza

Target virus subtype	Description of vaccine candidate	Adjuvant	Findings	Reference
H9N2	Inactivated whole virus (A/HK/1073/99). 7.5, 3.8, 1.9 μg/dose with adjuvant or 15 μg without adjuvant. 2 doses, day 0 and day 21	Aluminum hydroxide	Two doses needed to achieve HI* antibody titer of >1:40 at any dose.	(*22*)
H9N2	H9N2 whole virus or subunit vaccine. 7.5, 15, or 30 μg per dose. 2 doses, day 0 and day 21.	None	Two doses needed to achieve HI titer of >1:40 in persons <32 years of age; 1 dose needed to achieve HI titer of ≥1:40 in persons >32 y of age.	(*23*)
H5N1	Low pathogenicity H5N3 strain (A/duck/Singapore/F119-3/97) subunit vaccine with or without adjuvant. 7.5, 15, 30 μg per dose. 2 doses, day 0, day 21	MF59	Geometric mean antibody and seroconversion rates significantly higher when vaccine administered with adjuvant; 2 doses of vaccine needed to achieve antibody responses indicative of protection.	(*24*)
H5N1	Purified baculovirus-expressed recombinant H5 HA derived from A/HK/156/97. 25, 45, 90 μ g per dose, 2 doses or 1 dose of 90 μg followed by 10-μg dose	None	23% of volunteers had neutralizing titers of >1:80 after a single dose of 90 μg; 52% of volunteers had neutralizing antibody titers after 2 doses of 90 μg.	(*27*)

*HI, hemagglutination inhibition.

### Generating Vaccine Seed Viruses

The interpandemic period must be used to explore the optimal scientific, manufacturing, regulatory, and clinical research strategies for developing vaccines that are effective against pandemic influenza so that a vaccine will be available as soon as possible in the event of a pandemic. To this end, the Laboratory of Infectious Diseases, National Institute of Allergy and Infectious Diseases (NIAID), National Institutes of Health (NIH), is embarking on a program to develop candidate vaccines to prevent influenza pandemics caused by AI viruses. The vaccine seed viruses to be generated are based on the live attenuated cold-adapted influenza virus vaccines developed by Maassab and colleagues at the University of Michigan in the 1960s (*28*) and used as the basis for the FluMist vaccine (MedImmune, Inc., Gaithersburg, MD, USA) now licensed in the United States for persons 5–49 years of age for preventing interpandemic influenza. The principles of the development of such vaccines and safety and efficacy studies conducted in humans are reviewed elsewhere (*29**,**30*). The vaccine seed virus development strategy is not exclusive to live, attenuated vaccines, and similar studies with inactivated vaccines against different AI subtypes should be initiated.

The goal of our research program is to establish the "proof of principle" that the A/AA/6/60 cold-adapted (AA ca) virus bearing AI virus HA and NA genes will be infectious, immunogenic, and safe in humans and therefore of potential use for controlling pandemic influenza. The observed efficacy of live, attenuated vaccines for human interpandemic influenza, together with the findings to date that inactivated or subunit AI vaccines are suboptimally immunogenic in humans, strongly suggests that using live vaccines against pandemic influenza is worth exploring. Live, attenuated AI vaccines might require fewer doses and might provide broader immune responses than inactivated or subunit vaccines.

Live, attenuated influenza A candidate vaccines bearing the 6 internal genes of the AA ca donor virus (the attenuating genes) and the 2 protective HA and NA genes from human H3 or H1 viruses have been studied extensively in humans and have been licensed for general use. These vaccines are safe, infectious, immunogenic, nontransmissible, genetically stable, and efficacious (reviewed in [30]). It is reasonable to propose that a live, attenuated vaccine would rapidly induce protective immune responses, but this requires experimental verification in humans.

The pandemic influenza vaccine candidates will be generated by plasmid-based reverse genetics, shown in the Figure, panel A (reviewed in [31]). This technique allows infectious virus to be recovered from cells approved for use in human vaccine development (so-called qualified cells). These cells are cotransfected with plasmids encoding each of the 8 influenza gene segments to generate recombinant viruses that contain the HA and NA genes from AI viruses and 6 internal gene segments from the AA ca virus (*31*). Reverse genetics will allow modification of known virulence motifs in the HA or NA genes, such as the removal of the multibasic amino acid cleavage site motif in the HA gene of highly pathogenic AI strains that is associated with virulence in birds (*32*). The other advantage of reverse genetics is that a selection system is not needed to derive appropriate reassortant viruses from a background of parental viruses. In addition, the plasmids encoding the genes from the attenuated vaccine donor strain are available, and only the HA and NA genes need to be cloned for each vaccine. Several H5N1 vaccine candidates have been developed by using this technique (*33**–**36*). Some potential obstacles to applying the reverse genetics approach include the need for qualified cells for virus production and intellectual property for this technique. However, as long as the HA and NA gene segments do not have to be modified, the 6-2 gene reassortant investigational pandemic vaccines can be generated by genetic reassortment, as shown in the Figure, panel B. A candidate H9N2 pandemic vaccine was generated by using this technique (*37*).

**Figure:**
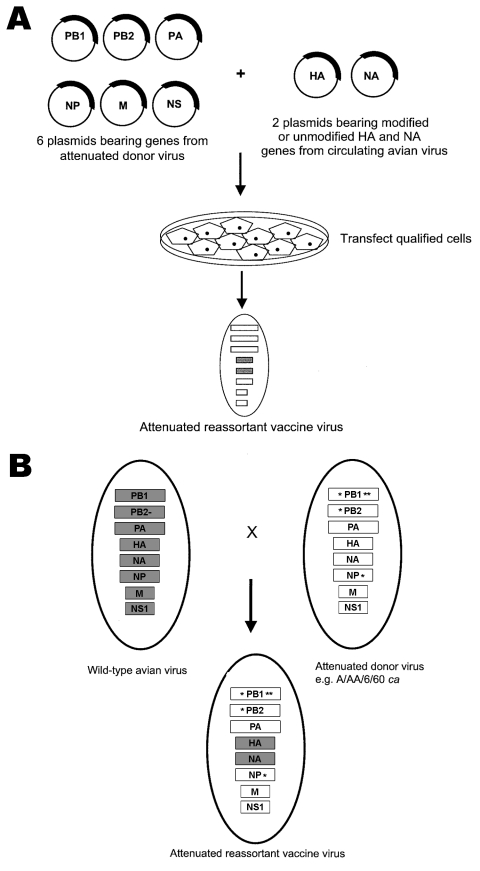
A) The 8-plasmid reverse genetics system to generate recombinant, live, attenuated pandemic influenza vaccines. Six plasmids encoding the internal genes of the attenuated donor virus are mixed with 2 plasmids encoding the circulating avian virus hemagglutinin (HA) and neuraminidase (NA) genes (which may or may not have been modified to remove virulence motifs). Qualified cells are transfected with the plasmids, and the attenuated reassortant virus is isolated. B). Generation of live, attenuated pandemic influenza vaccine viruses with the 6 internal genes from the attenuated donor virus bearing attenuating mutations (*) and the HA and NA genes from the circulating avian virus by classic reassortment. The 6-2 reassortants generated by this method are selected in the presence of antiserum specific for HA and NA of the attenuated donor virus.

Live, attenuated vaccines must be able to replicate to levels that elicit a protective immune response without causing disease in the host, so a balance of infectivity, level of attenuation, and immunogenicity must be achieved. Therefore, before the next pandemic, we must evaluate in humans the safety, infectivity, immunogenicity, and phenotypic stability of live, attenuated influenza A candidate vaccines. The types of in vitro and in vivo studies that will be performed before clinical trials in humans are initiated, in addition to standard safety tests, are listed in Table 4. In vitro studies will be performed to confirm the genome sequence of the vaccine viruses. The cold-adapted and temperature-sensitive phenotype of the vaccine viruses will be confirmed in vitro in tissue culture. The attenuation phenotype of the vaccine candidates must be tested in an appropriate animal model. A critical step in evaluating vaccine candidates is selecting a model in which restriction of replication of the vaccine virus can be convincingly demonstrated in comparison to the wild-type parent virus. Since we cannot predict how AI viruses of different subtypes will behave in different animal species, animal models for each virus subtype will be developed. The use of rodent models (e.g., mice and hamsters) will be explored. The use of a ferret model will be investigated as well, although limited availability of influenza-seronegative ferrets and facilities in which highly pathogenic wild-type AI viruses can be evaluated in ferrets makes such studies logistically and practically difficult for assessing large numbers of candidate vaccines. In addition, the higher body temperature of the ferret may confound interpretation of studies in which replication of temperature-sensitive viruses is being assessed. The vaccine viruses may also require evaluation in the standard Office International des Épizooties (World Organization for Animal Health) intravenous pathogenicity test in chickens to confirm that they are not highly pathogenic in chickens and, as such, do not pose a threat to the poultry industry. Such a requirement will be guided by national agricultural authorities. Immunogenicity, dose response, antibody response kinetics, and efficacy studies will also be carried out in appropriate animal models before clinical trials.

**Table 4:** Preclinical testing to be performed on live attenuated pandemic influenza vaccine candidates

In vitro testing	In vivo testing
Confirmation of virus genome sequence Trypsin-dependent replication in cell culture Confirmation of phenotype associated with the vaccine donor virus, e.g., temperature sensitivity, cold adaptation	Intravenous pathogenicity test in chickens Attenuation (restricted replication) in rodent or ferret model Immunogenicity in rodent or ferret model Protective efficacy in rodent model

Past experience with live, attenuated vaccines for interpandemic human influenza (*30*) indicates that live virus vaccines may have great potential for use as vaccines during pandemic spread of influenza because of their high level of immunogenicity for immunologically naive persons and their ability to rapidly induce immunity, i.e., within the first 10 days after vaccination. The contribution of cellular immune responses to the control of AI virus infection remains to be determined and can be examined in the context of live, attenuated vaccines. Such responses may be valuable in an influenza pandemic, in which the vaccine may protect from severe illness or death even if it is not completely antigenically matched to the emergent strain. Since a live, attenuated virus vaccine based on the AA ca donor virus has been licensed by the Food and Drug Administration for general use in healthy persons 5–49 years of age, the infrastructure for manufacture and characterization of live, attenuated virus vaccines exists. The availability of the manufacturing capability for a live, attenuated virus vaccine makes it feasible to initiate a project in collaboration with industry to develop seed viruses for live attenuated vaccines against influenza A viruses with pandemic potential.

Our overall plan includes the following steps: 1) generation of a set of live, attenuated viruses bearing an H4–H16 HA and the accompanying NA found in the wild-type virus (we will not generate novel combinations of HA and NA proteins) on the attenuated AA ca donor virus background; 2) preparation and qualification of a clinical lot of each pandemic vaccine candidate; 3) evaluation of the safety, infectivity, immunogenicity, and phenotypic stability of each candidate vaccine in humans; 4) storage of human sera obtained from vaccinees to determine antigenic relatedness of the vaccine administered to the study participant with actual newly emerged pandemic viruses; and 5) storage of seed viruses for manufacture of vaccine to prevent disease caused by pandemic viruses that do emerge. Thus, vaccine manufacture can be initiated with pretested viruses without delay. Even if the seed virus does not match the pandemic strain and a vaccine virus that is an exact match has to be generated, the dosing and immunogenicity data from the previous vaccine studies can guide its use. If the AA ca reassortant virus is safe and attenuated but infectious in humans, it can be used as a challenge virus to assess vaccine efficacy for both live and inactivated influenza virus vaccines.

A major concern associated with using a live, attenuated influenza vaccine bearing genes derived from an AI virus is the risk for reassortment of the vaccine virus with a circulating influenza virus. This reassortment could result in a novel subtype of influenza that could spread in the human population. Although such an event may not be of concern in the face of widespread disease from a pandemic strain of influenza, it would clearly be an unfavorable outcome if the threatened pandemic did not materialize. Clinical trials in humans of these live, attenuated pandemic vaccine candidates will be performed in carefully planned and executed inpatient studies. The risk for reassortment must be carefully considered by public health authorities before a decision is made to introduce a live, attenuated vaccine in a threatened pandemic. Using every available option to develop vaccines that may be used for an influenza pandemic is critical.

## Conclusions

Recent events in Asia have led to intensive planning and preparation for a potential global influenza pandemic. Vaccine development is a critical part of preparedness. Recent studies that used mathematical models to study potential intervention strategies predicted that local prevaccination with a vaccine that is 70% efficacious against the pandemic strain could enhance the effectiveness of antiviral prophylaxis in preventing spread of the virus (*38*). Production and establishment of the proof of principle of candidate live and inactivated vaccines with AI HA and NA proteins in the interpandemic period could save valuable time in the event of a pandemic. Such studies will also provide information about the biology of AI viruses and immune responses to them in humans.
